# Correction: A spatio-temporal approach to short-term prediction of visceral leishmaniasis diagnoses in India

**DOI:** 10.1371/journal.pntd.0010346

**Published:** 2022-04-04

**Authors:** The PLOS NTDs Staff

S3 Fig is a duplicate of S5 Fig. Please view the correct S3 Fig below. The publisher apologizes for the error.

## Supporting information

S3 Fig PIT histograms for selected models at each stage. Model 42 is the final model. Model 52 offered minor improvement in RPS with additional complexity.
(TIF)
