# Supplementary material for: A spatio-temporal approach to short-term prediction of visceral leishmaniasis diagnoses in India
Source: PLoS Negl Trop Dis. 2020 Jul 9;14(7):e0008422. doi: 10.1371/journal.pntd.0008422 (PMC7373294; doi:10.1371/journal.pntd.0008422)
Supplement: S1 Text — (PDF) [file pntd.0008422.s001.pdf]

Supporting Information

S1 Text

Model Selection

Starting with a basic, endemic-only model (including a population offset and linear trend in time), potential extensions of the three core components were added in turn and measures of fit and predictive power were calculated. The addition which yielded the best improvement in the RPS of OSA predictions, subject to calibration (p not less than 0.1 for test of calibration based on RPS), was selected and then all remaining options tested again. This process was repeated until no further extension of the model made a significant ($p < 0.001$) improvement to predictive power (as determined by a permutation test on the RPS). This stringent criterium was employed in order to prioritise simplicity over complexity. If at any point an individual model parameter lost significance, the element associated with this parameter was removed in subsequent models.

Empirical Coverage Probabilities

Again using a one-step-ahead approach, the 25th and 75th quantiles of the predicted distribution were calculated and a score of 0 or 1 assigned if the observed value fell inside or outside this quantile range respectively. This binary score was assigned for each block and each month in the test set, such that we could subsequently calculate a proportion of prediction intervals which did not capture the true count. Thus, the overall score, C, is given by

$$C = \frac{1}{n_i n_t} \sum_{i,t} \mathbb{1}[y_{it} \leq q_{i,t,0.25} | y_{it} \geq q_{i,t,0.75}] \tag{1}$$

where y_{it} is the observed count for block i at month t, n_i and n_t the total number of blocks and months respectively, and $q_{i,t,p}$ the pth quantile of the predicted distribution. We also investigated such a score using 10th and 90th quantiles, to ascertain whether

these could be used as approximate lower and upper bounds for case counts.
