# Supplementary material for: A spatio-temporal approach to short-term prediction of visceral leishmaniasis diagnoses in India
Source: PLoS Negl Trop Dis. 2020 Jul 9;14(7):e0008422. doi: 10.1371/journal.pntd.0008422 (PMC7373294; doi:10.1371/journal.pntd.0008422)
Supplement: S2 Text — (PDF) [file pntd.0008422.s002.pdf]

Supporting Information

S2 Text

Preliminary analyses

Dispersion

District-specific dispersion parameters were investigated, but ultimately not considered a viable option to be included in the model. Four districts in particular (Aurangabad, Banka, Jehanabad and Nawada) demonstrate extended periods of zero incidence with occasional sporadic cases or large spikes, which lead to very large dispersion estimates for these districts and therefore unrealistically high predictions. See S1 Fig. for an illustration of these patterns. Due to the neighbourhood effect, these high predictions in turn influence the predictions of any bordering blocks. Changes in detection effort could go some way to explaining these unusual patterns, however it is also likely that such patterns will become more common as elimination is approached. This suggests that an alternative modelling strategy will become necessary as cases become more sparse in space and time.

Distributed temporal lags

By sequentially adding further distributed lags to the best-fitting single-lagged model, neither a clear minimum nor an "elbow" in RPS was attained up to twelve months. The weights assigned to each lag did not show a rapid "drop-off" as a result of a high estimated decay parameter, and months substantially far back in time were still assigned non-negligible weight. PIT histograms of predictions from these lagged models are included in S2 Fig. We found that adding higher orders of distributed lags consistently improved both predictive power and fit. This appears to contradict analysis of individual block time series which suggested significant auto-correlation no more than four months back in time. In the current form of "hhh4addon", it is not possible to specify a different temporal lag length within the AR and NE components (for example,

to incorporate neighbouring incidence from further back in time than within-block). Therefore, the contribution of distributed lags to both components had to be considered and a balance had to be drawn. Comparing the PIT histograms of solely auto-regressive models, the very highest counts are vastly underestimated for all lag lengths. Since the highest values in each block often reflect sudden jumps they cannot be captured by auto-regression; more information - potentially from the surrounding area - is required to anticipate them. Models with no auto-regression but which incorporate neighbouring incidence are better able to reach the highest counts but in doing so over-estimate the moderate-to-high range. It was concluded that beyond four months of lags the improvement in prediction was small enough to discount, and much longer lags were difficult to justify epidemiologically. Therefore only four months of lags were considered for the final model.
