# Supplementary figures and images for: A spatio-temporal approach to short-term prediction of visceral leishmaniasis diagnoses in India
Source: PLoS Negl Trop Dis. 2020 Jul 9;14(7):e0008422. doi: 10.1371/journal.pntd.0008422 (PMC7373294; doi:10.1371/journal.pntd.0008422)

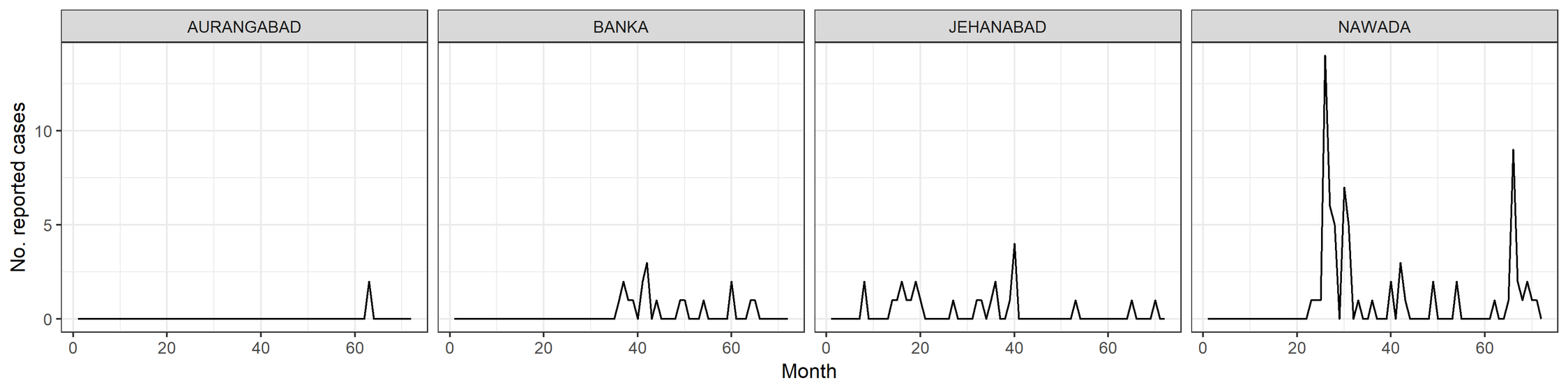

Supplement: S1 Fig — (TIF) [file pntd.0008422.s003.tif]

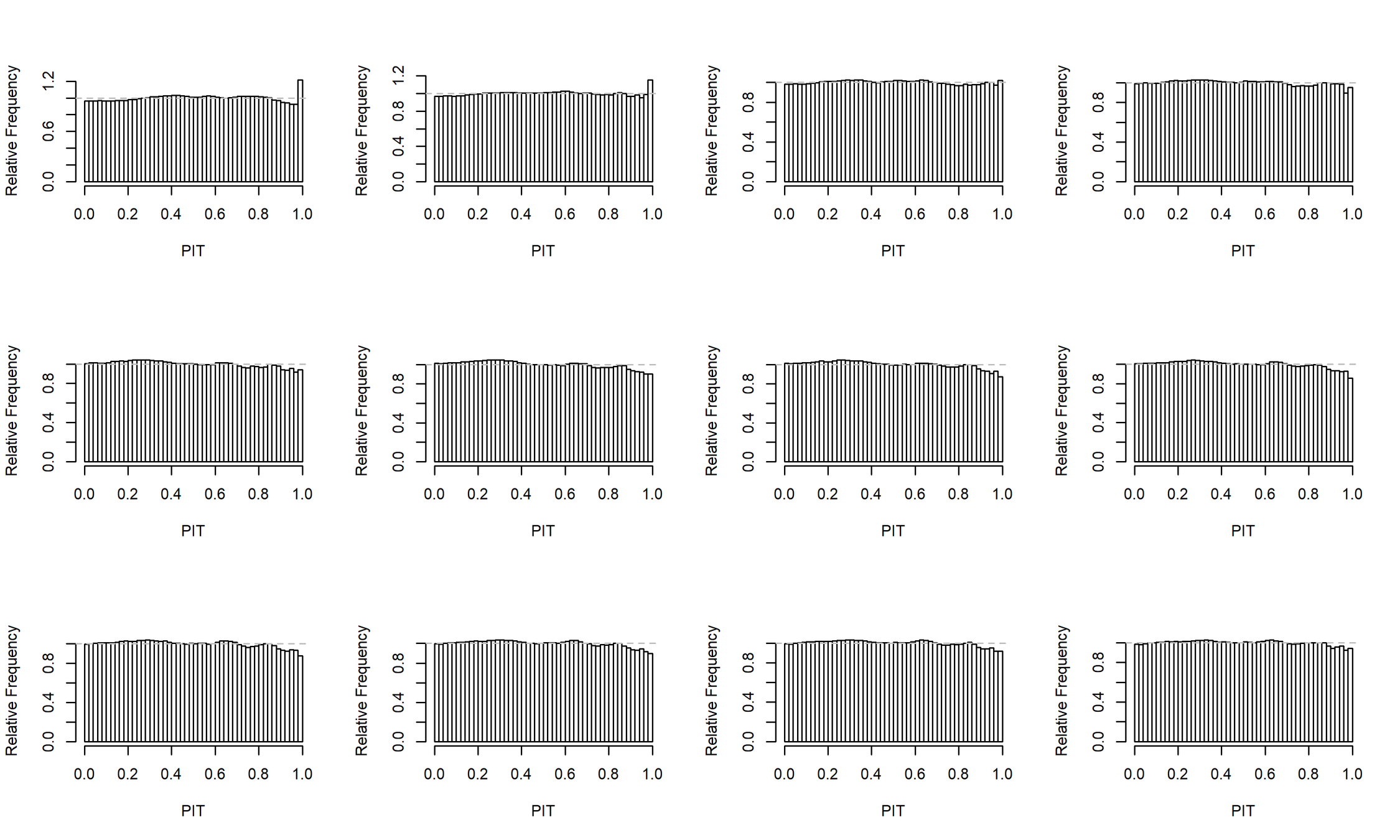

Supplement: S2 Fig — The final model selection process considered up to four lags. (TIF) [file pntd.0008422.s004.tif]

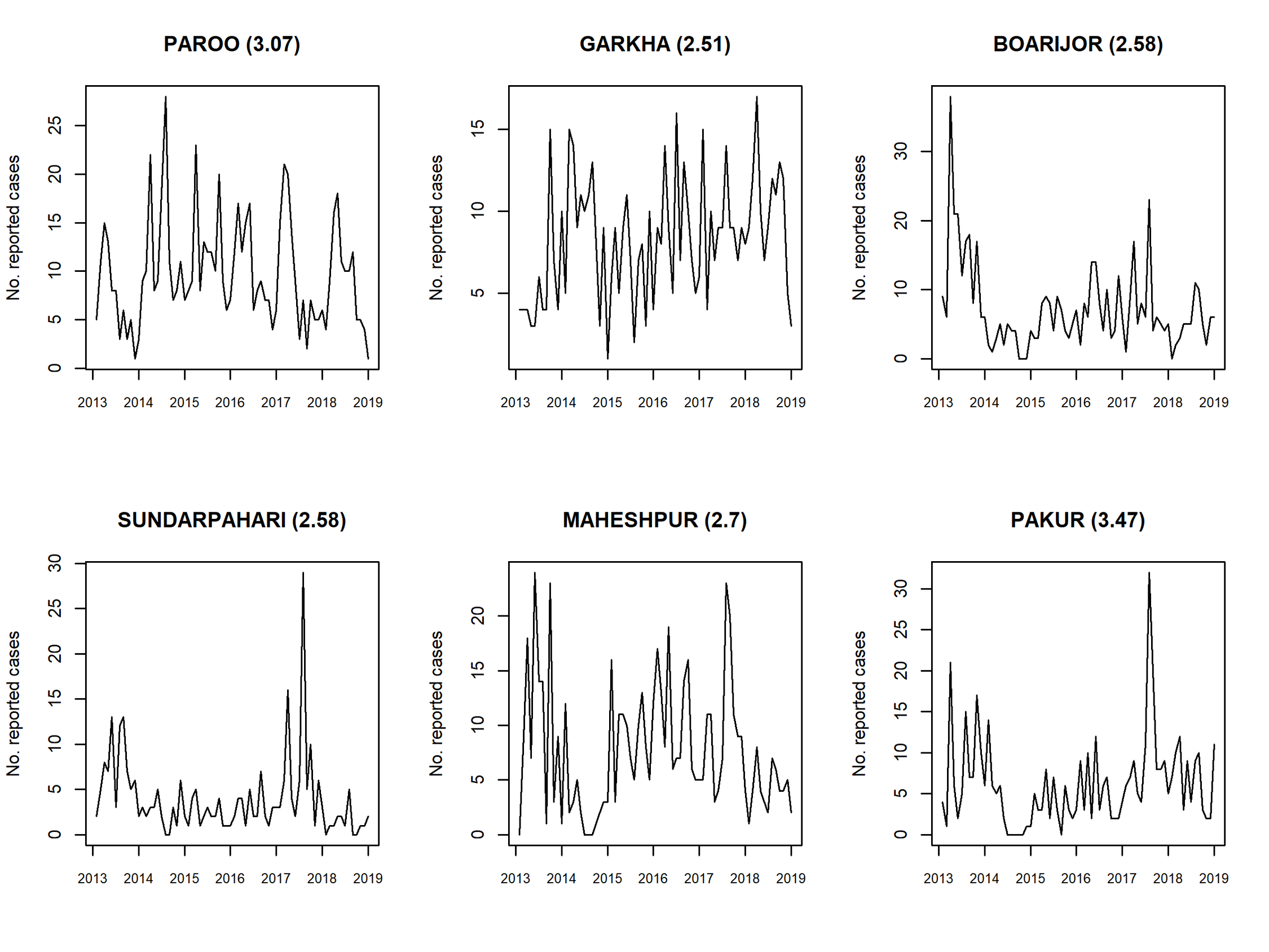

Supplement: S3 Fig — Model 42 is the final model. Model 52 offered minor improvement in RPS with additional complexity. (TIF) [file pntd.0008422.s005.tif]

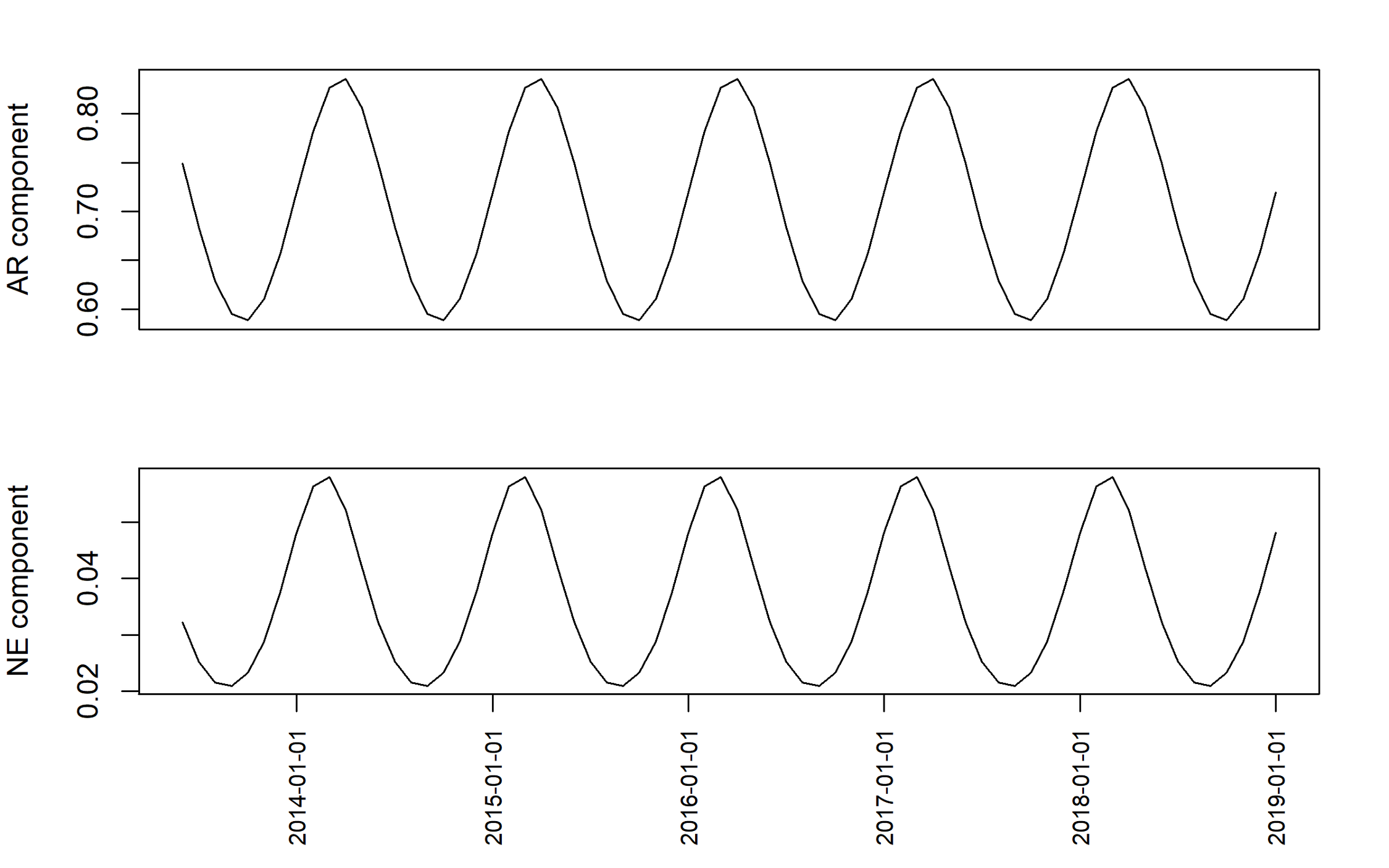

Supplement: S4 Fig — Both reflect the first-quarter peak in reported cases but the magnitude of the waves differs, with the contribution of the AR component varying more than that of the NE. (TIF) [file pntd.0008422.s006.tif]
