# Supplementary material for: A spatio-temporal approach to short-term prediction of visceral leishmaniasis diagnoses in India
Source: PLoS Negl Trop Dis. 2020 Jul 9;14(7):e0008422. doi: 10.1371/journal.pntd.0008422 (PMC7373294; doi:10.1371/journal.pntd.0008422)
Supplement: S1 Table — The value of S within the formula indicates the number of seasonal waves included. The reported AIC is for the fit to training data only, and RPS is of predictions made without updating this fit (i.e. fixed instead of rolling). C2575 and C1090 refer to the coverage of 50% and 80% quantile intervals, respectively, alongside the average interval width in cases. Model no. 42 is the final model. (PDF) [file pntd.0008422.s008.pdf]

S1 Table

Fit and prediction metrics for selected model at each stage. The value aof S within the formula indicates the number of seasonal waves included. The reported AIC is for the fit to training data only, and RPS is of predictions made without updating this fit (i.e. fixed instead of rolling). C2575 and C1090 refer to the coverage of 50% and 80% quantile intervals, respectively, alongside the average interval width in cases. Model no. 42 is the final model.

Stage	Model No.	END	AR	NE	Dispersion	No. parameters	AIC	RPS	Calibration (p-value)	C1090	Avg. width
0	1	offset + 1 + t			1	3	65412	0.657	<0.0001	0.095	2.243
1	2	offset + 1 + t + seas(∼1, S=1)			1	5	65227	0.654	<0.0001	0.090	2.330
1	3	offset + 1			1	2	65811	0.698	<0.0001	0.044	4.158
1	4	offset + 1 + t + logpopdens			1	4	65708	0.662	<0.0001	0.094	2.180
1	5	offset + 1 + t	AR(1)		1	4	57100	0.495	0.109	0.060	2.386
**1**	**6**	**offset + 1 + t**	**AR(1) + seas(∼1, S=1)**		**1**	**6**	**57058**	**0.493**	**0.115**	**0.058**	**2.388**
1	7	offset + 1 + t	AR(1) + seas(∼1 + t, S=1)		1	7	57031	0.496	<0.0001	0.064	2.171
1	8	offset + 1 + t		NE(2)	1	5	56755	0.516	0.003	0.056	2.304
1	9	offset + 1 + t		NE(2) + logpopdens	1	5	56763	0.516	0.002	0.056	2.313
1	10	offset + 1 + t		NE(2) + seas(∼1, S = 1)	1	7	56685	0.515	0.001	0.054	2.308
1	11	offset + 1 + t		NE(2) + seas(∼1 + t, S = 1)	1	8	56680	0.516	0.203	0.057	2.201
1	12	offset + 1 + t			State	4	65310	0.659	<0.0001	0.098	2.145
2	13	offset + 1 + seas(∼1, S=1)	AR(1) + seas(∼1, S=1)		1	7	57024	0.502	<0.0001	0.048	2.627
2	14	offset + 1	AR(1) + seas(∼1, S=1)		1	5	57101	0.502	<0.0001	0.049	2.612
2	15	offset + 1 + t + logpopdens	AR(1) + seas(∼1, S=1)		1	6	57128	0.499	<0.0001	0.055	2.496
2	16	offset + 1 + t	AR(1) + seas(∼1 + t, S=1)		1	7	57031	0.496	<0.0001	0.064	2.171
2	17	offset + 1 + t	AR(1) + seas(∼1 + t, S=2)		1	9	56996	0.496	<0.0001	0.064	2.176
2	18	offset + 1 + t	AR(1) + seas(∼1, S=1)	NE(2)	1	8	53362	0.458	0.210	0.055	2.105
2	19	offset + 1 + t	AR(1) + seas(∼1, S=1)	NE(2) + seas(∼1, S = 1)	1	10	53300	0.457	0.294	0.053	2.101
2	20	offset + 1 + t	AR(1) + seas(∼1, S=1)	NE(2) + seas(∼1 + t, S = 1)	1	11	53301	0.458	0.125	0.053	2.122
2	21	offset + 1 + t	AR(1) + seas(∼1, S=1)	NE(2) + logpopdens	1	8	53398	0.458	0.144	0.054	2.111
2	22	offset + 1 + t	AR(1) + seas(∼1, S=1)		State	7	57059	0.493	0.123	0.058	2.389
**2**	**23**	**offset + 1 + t**	**AR(2) + seas(∼1, S=1)**		**1**	**6**	**53833**	**0.455**	**0.189**	**0.053**	**2.230**
2	24	offset + 1 + t	AR(3) + seas(∼1, S=1)		1	6	52279	0.439	0.005	0.061	2.017
2	25	offset + 1 + t	AR(4) + seas(∼1, S=1)		1	6	51342	0.428	<0.0001	0.064	1.877
3	26	offset + 1 + seas(∼1, S=1)	AR(2) + seas(∼1, S=1)		1	7	53806	0.457	<0.0001	0.043	2.395
3	27	offset + 1	AR(2) + seas(∼1, S=1)		1	5	53844	0.458	<0.0001	0.047	2.340

Stage	Model No.	END	AR	NE	Dispersion	No. parameters	AIC	RPS	Calibration (p-value)	C1090	Avg. width
3	28	offset + 1 + t + logpopdens	AR(2) + seas(∼1, S=1)		1	6	53835	0.456	<0.0001	0.042	2.404
3	29	offset + 1 + t	AR(2) + seas(∼1 + t, S=1)		1	7	53815	0.455	0.002	0.056	2.087
3	30	offset + 1 + t	AR(2) + seas(∼1 + t, S=2)		1	9	53692	0.455	0.001	0.057	2.079
3	31	offset + 1 + t	AR(3) + seas(∼1, S=1)		1	6	52279	0.439	0.005	0.061	2.017
3	32	offset + 1 + t	AR(2) + seas(∼1, S=1)	NE(1)	1	7	51749	0.437	0.181	0.054	1.974
**3**	**33**	**offset + 1 + t**	**AR(2) + seas(∼1, S=1)**	**NE(1) + seas(∼1, S = 1)**	**1**	**9**	**51675**	**0.437**	**0.122**	**0.055**	**1.966**
3	34	offset + 1 + t	AR(2) + seas(∼1, S=1)	NE(3) + seas(∼1 + t, S = 1)	1	11	51543	0.437	0.656	0.050	2.029
3	35	offset + 1 + t	AR(2) + seas(∼1, S=1)		State	7	53831	0.455	0.192	0.053	2.230
4	36	offset + 1 + seas(∼1, S=1)	AR(2) + seas(∼1, S=1)	NE(1) + seas(∼1, S = 1)	1	10	51701	0.437	0.085	0.056	1.961
4	37	offset + 1	AR(2) + seas(∼1, S=1)	NE(1) + seas(∼1, S = 1)	1	8	51673	0.437	0.194	0.055	1.969
4	38	offset + 1 + t + logpopdens	AR(2) + seas(∼1, S=1)	NE(1) + seas(∼1, S = 1)	1	9	51691	0.437	0.153	0.056	1.962
4	39	offset + 1 + t	AR(2) + t	NE(1) + seas(∼1, S = 1)	1	8	51670	0.439	0.001	0.059	1.865
4	40	offset + 1 + t	AR(2) + seas(∼1, S=2)	NE(1) + seas(∼1, S = 1)	1	11	51545	0.437	0.115	0.055	1.973
4	41	offset + 1 + t	AR(2) + seas(∼1 + t, S=2)	NE(1) + seas(∼1 + t, S = 1)	1	15	51446	0.441	0.563	0.054	1.959
**4**	**42**	**offset + 1**	**AR(4) + seas(∼1, S=1)**	**NE(1) + seas(∼1, S = 1)**	**1**	**8**	**50323**	**0.420**	**0.346**	**0.054**	**1.872**
4	43	offset + 1 + t	AR(2) + seas(∼1, S=1)	NE(1) + t	1	8	51749	0.437	0.545	0.053	2.003
4	44	offset + 1 + t	AR(2) + seas(∼1, S=1)	NE(1) + seas(∼logpopdens, S = 1)	1	9	51780	0.438	0.202	0.056	1.975
4	45	offset + 1 + t	AR(2) + seas(∼1, S=1)	NE(3)	1	8	51642	0.437	0.333	0.053	1.972
4	46	offset + 1 + t	AR(2) + seas(∼1, S=1)	NE(1) + seas(∼1, S = 1)	State	10	51676	0.437	0.118	0.055	1.964
4	47	offset + 1 + t	AR(2) + seas(∼1, S=1)	NE(1) + seas(∼logpopdens + t, S = 1)	State	11	51782	0.438	0.314	0.055	1.988
5	48	offset + 1 + seas(∼1, S=1)	AR(4) + seas(∼1, S=1)	NE(1) + seas(∼1, S = 1)	1	10	50342	0.420	0.297	0.055	1.867
5	49	offset + 1	AR(4) + seas(∼1 + t, S=1)	NE(1) + seas(∼1 + t, S = 1)	1	10	50296	0.424	0.614	0.052	1.864
5	50	offset + 1 + logpopdens	AR(4) + seas(∼1, S=1)	NE(1) + seas(∼1, S = 1)	1	9	50332	0.420	0.439	0.054	1.870
5	51	offset + 1	AR(4) + t	NE(1) + seas(∼1, S = 1)	1	7	50336	0.424	0.000	0.060	1.763
**5**	**52**	**offset + 1**	**AR(4) + seas(∼1, S=2)**	**NE(1) + seas(∼1)**	**1**	**10**	**50164**	**0.419**	**0.194**	**0.055**	**1.868**
5	53	offset + 1	AR(4) + seas(∼1 + t, S=1)	NE(1) + seas(∼1 + t, S = 2)	1	14	50097	0.423	0.782	0.052	1.851
5	54	offset + 1	AR(4) + seas(∼1, S=1)	NE(1) + seas(∼1 + t, S = 1)	1	9	50324	0.420	0.620	0.052	1.904
5	55	offset + 1	AR(4) + seas(∼1, S=1)	NE(1) + seas(∼logpopdens, S = 1)	1	8	50401	0.421	0.425	0.055	1.873
5	56	offset + 1	AR(4) + seas(∼1, S=1)	NE(1)	1	6	50416	0.420	0.251	0.054	1.877
5	57	offset + 1	AR(4) + seas(∼1, S=1)	NE(1) + seas(∼1, S = 1)	State	9	50325	0.420	0.342	0.054	1.873
5	58	offset + 1	AR(4) + seas(∼1, S=1)	NE(1) + seas(∼logpopdens + t, S = 1)	State	10	50405	0.421	0.537	0.055	1.876
