# Supplementary material for: A spatio-temporal approach to short-term prediction of visceral leishmaniasis diagnoses in India
Source: PLoS Negl Trop Dis. 2020 Jul 9;14(7):e0008422. doi: 10.1371/journal.pntd.0008422 (PMC7373294; doi:10.1371/journal.pntd.0008422)
Supplement: Attachment — Submitted filename: Reviewer_comments_final.docx [file pntd.0008422.s009.docx]

The Editors,

PLoS NTDs

11th April 2020

**Re: A spatio-temporal approach to short-term prediction of visceral leishmaniasis diagnoses in India**

Dear Editors,

We would like to thank the reviewers for their comments on the above manuscript; our proposed amendments and responses are laid out below.

We have changed the manuscript significantly to address the reviewers’ comments and believe that it has made a substantial improvement.

Reviewer's Responses to Questions

Reviewer #1: The objectives of the study are not clearly articulated as the manuscript read like a tutorial for using spatio temporal models to produce and evaluate forecasts.

The primary objective is highlighted in line 3 and more fully articulated in lines 32-34 (beginning: “*The primary aim of this paper is to demonstrate a methodology for providing short-term, block-level predictions of VL cases for two purposes*.”). We have also added a more explicit hypothesis at the end of the introduction:

Line 169: “*Our hypothesis is that models constructed with the surveillance framework to accommodate spatio-temporal correlation in disease incidence can provide significantly more accurate (in terms of sharpness and calibration) predictions than a purely parameter-driven (i.e. independent of history and spatial context) model with overall mean and linear time trend.”*

We then draw a conclusion on this hypothesis in the discussion:

Line 385: “*We have presented the evaluation of a predictive model of VL in Bihar and four endemic districts in Jharkhand, demonstrating a substantial (36% lower RPS) benefit from incorporating spatial and historical case information when compared to a non-spatial, linear trend model.”*

The study fails to properly address the previous literature on leishmaniasis forecasts, for example (just looking at PLoS NTDs):

[https://journals.plos.org/plosntds/article?id=10.1371/journal.pntd.0003283](https://protect-eu.mimecast.com/s/aZPFCJQBwUXgPqcmT43M?domain=journals.plos.org)

[https://journals.plos.org/plosntds/article?id=10.1371/journal.pntd.0005353](https://protect-eu.mimecast.com/s/57jhCKQDxUJZ02TyvWFH?domain=journals.plos.org)

[https://journals.plos.org/plosntds/article?id=10.1371/journal.pntd.0000033](https://protect-eu.mimecast.com/s/5RQVCLJEyfvl5RHGNPvf?domain=journals.plos.org)

We thank the reviewer for highlighting these publications. We were aware of them but did not include them because they did not relate directly to our method or our subject. We have now included them, and have added reference to them in section 1.2 (lines 42, 61 63) and 1.4 (line 134). We have also included other recent publications on leishmaniasis forecasting:$Type equation here.$

Line 46: Li (2020) Predicting the number of visceral leishmaniasis cases in Kashgar, Xinjiang, China using the ARIMA-EGARCH model

Line 61: Amro (2020) Epidemiology and spatiotemporal analysis of visceral leishmaniasis in Palestine from 1990 to 2017

Line 68: Godana (2019) Dynamic spatiotemporal modeling of the infected rate of visceral leishmaniasis in human in an endemic area of Amhara regional state, Ethiopia

Line 88: Adegboye (2017) Spatially correlated time series and ecological niche analysis of cutaneous leishmaniasis in Afghanistan

No clear hypothesis is being tested about the forecasts, for example, a relevant question not addressed is how would nested simplified models perform when compared with the best model in the s1 table. To what extent is the spatial or temporal component really necessary for an accurate prediction? This is related with the issue that model search was not fully systematic (see lines 464-471). Authors could have tried model selection, at least, about the local AIC minima and the models with best prediction performance.

We believe that this comment arises from a misunderstanding of our approach. To add clarification, we have included the full table of tested models as an additional appendix and added the following to the results section:

Line 338: “*The final model achieved an overall RPS for one-step-ahead prediction of 0.420, 36% lower than the null (non-spatial and non-autoregressive) model and 8% lower than the best non-spatial model, with individual block-wise averages ranging from* $4.3*{10}^{-5}$ *to 3.47. This equates to a mean absolute error of 0.58, a 30% reduction from the null model.”*

We began with a simple model and expanded upon it, therefore the best model highlighted in table S1 demonstrated significantly better predictive performance (by permutation test on the RPS) than the simplest, “null” model nested within it. As can be seen from the extended table, the addition of spatial and temporal components (NE and AR) yielded improvement in both prediction score and AIC.

We have amended line 176 to specify that the model selection approach was *semi-*systematic forward-selection.

Methodologically, it is not clear why authors included time in both the temporal and spatial parts of the model.

We understand that this comment could relate to one of two possible points: that - alongside the auto-regressive component - the spatial component in the model is also lagged in time, or that a time-varying effect (seasonal forcing) is included in the coefficients of both the spatial and temporal components. In either case we believe that this is included in the paper. We can we address this issue if we have misunderstood.

If the former, we explain in the original description of the surveillance framework (cited on line 87) and we also direct the reader to another application in which this structure has been used (line 88) we explain that we would expect a force of infection coming from neighbouring regions to be lagged in time; that is to say, due to the disease’s generation and detection time, transmission in one region would be influenced by the number of cases in the surrounding regions some months previously.

If the latter, we aimed to take an objective view and seek the model structure which yielded best predictive power, without a priori assumptions about what might be appropriate given the disease’s epidemiology. Although it is perhaps not straightforward to interpret mechanistically, seasonal forcing in the coefficients of both the AR and NE components was found to significantly improve overall predictive power. The contribution of seasonal forcing is explained in lines 315-325.

Related to this, it is also unclear why the authors try to propose new terms (autocorrelation, neighborhood and endemic) for what has been traditionally referred as temporal, spatial and external (also exogenous or extrinsic) in the time series literature and in the theory for nonlinear dynamic models (e.g., Kaplan and Glass 1997) that underlies the type of model fitted.

We do not propose these new terms– they are used by the authors of the framework we used and are established in the field from which it originates. We did not want to confuse the reader by using multiple terms for the same concepts, or terms which did not match to the package’s source material. The framework in *surveillance* is based upon a multivariate TSIR model [Section 1.4.2, Handbook of Infectious Disease Data Analysis, Wakefield 2019].

Related the temporal auto-correlation the selection of sine and cosine functions is known to be prone to grossly misrepresent the seasonality of environmentally forced diseases, as seasonal patterns tend to be asymmetric and are more clearly described by seasonal autoregressive functions or via the decomposition of signals (See Priestley 1988, [https://books.google.co.cr/books/about/Non_linear_and_Non_stationary_Time_Serie.html?id=DvywVGExSZkC&redir_esc=y](https://protect-eu.mimecast.com/s/86ohCM1GzclvGqF4gR91?domain=books.google.co.cr)).

We agree. In this model framework, seasonality in the case counts is not directly characterized by the sine/cosine terms, but by autoregression with additionally seasonally varying strength (perhaps what the reviewer refers to as a “seasonal autoregressive function”). The sine/cosine terms appear as predictors for the *coefficients* of the autoregressive and spatially autoregressive model components, not as direct predictors of the log case count (linear predictor). We have attempted to clarify this in line 240

Line 240: “*Seasonal variation and linear trend within the coefficients of all three components, serving to vary the relative strength of each component over time*.”

As can be seen in the extended model table S1 ( models 17, 30, 40, 41, 52 and 53), seasonality with additional trigonometric terms (i.e. decomposed into $\frac{2\pi}{12}$ and $\frac{4\pi}{12}$ terms) were tested but ultimately not included in the final model.

In the models it is not clear why time was included both in the temporal and spatial part of the model, using the same functions and what was done to avoid problems of unidentifiability.

The first part of this comment is addressed above. With regards to the second part, we did not encounter any issues with identifiability of model parameters. Held and Paul (2012) ([https://onlinelibrary.wiley.com/doi/pdf/10.1002/bimj.201200037](https://protect-eu.mimecast.com/s/sYssCzXkjiYP11u4JhkA?domain=onlinelibrary.wiley.com)) conducted extensive simulation studies to show that the different seasonal terms are identifiable.

The part about model evaluation is also written in a highly mystified way, since at the end what is done testing whether the model predictions capture the peaks and throughs in cases.

We have added the following on line 344 to emphasise that model evaluation and comparison was made by mean RPS of predictions made across the whole 24-month test period:

Line 344: “*Model selection was performed based on the model’s mean RPS across all blocks and the whole test period (specifically 502 blocks * 24 months, so 12048 test predictions), but beneath this overall score is a broader distribution of scores for each block-month prediction which is influenced by peaks, troughs and otherwise unusual incidence patterns. The histogram in Fig. 6 illustrates the distribution over blocks, demonstrating that the final model is able to predict accurately and precisely across the majority of the region, yet there is a small subset of blocks with more widely varying RPS.”*

In this section (3.3.1) we therefore looked into which particular points in space and time were predicted best/worst (i.e. gave the lowest/highest scores) for the final model.

In line 115 it is worth checking that scoring rules were used in the suggested references above.

Proper scoring rules were not used in the suggested references; however, they were used in the cited publication which applies the *surveillance* method to analyse cutaneous leishmaniasis in Afghanistan [Adegboye (2016)]. Beyond this, proper scoring rules are broadly considered to be the absolute standard in probabilistic forecasting; see Held (2017) and Funk (2019) for examples of the use of RPS, while the log score is used in major forecasting challenges such as FluSight (<https://www.cdc.gov/flu/weekly/flusight/index.html>) and the White House Dengue Forecasting Project (<https://www.pnas.org/content/116/48/24268.short>). We have expanded our explanation of these benefits in lines 133-145:

*Line 133: “To assess performance of probabilistic forecasts it is standard to use proper scoring rules [Czado (2009), Gneiting (2014), Held (2017), Lu (2020), Funk (2019)], which offer more detailed scrutiny of the prediction than measures of absolute or squared error (as used, for example in Chaves (2007)) by taking into account the whole predicted distribution. In fact, the ranked probability score (RPS) can be considered a generalisation of absolute error, to which it reduces if the forecast distribution consists of a single point. Proper scoring rules measure simultaneously the calibration and sharpness of forecast distributions; they capture the model's ability to predict both accurately and precisely but also to identify its own uncertainty in that prediction Funk (2019). With a well-calibrated model the observed values should appear as having come from the predicted distribution at that point, and we want as precise or sharp a predicted distribution as possible while maintaining that calibration. In contrast, the mean absolute error for example only evaluates how well the central tendency of predictions aligns with the observations.”*

In Lines 196-200 the comparison of rolling windows has been previously used in leishmaniasis forecasts (see suggested references above)

We thank the reviewer for highlighting this and have added the reference in lines 229 and 270.

Reviewer #2: With this paper being about the development and assessment of a model, the methods (with ample information needed from the background section) well described the model development process and implementation for developing potential models to compare for the best potential predictor. This was very mathematically complicated, but I think covered well their development of the model. There was no stated "hypothesis" per se, but essentially they are looking at among the models made, what is the best model to predict future cases.

The reviewer’s interpretation of the hypothesis is correct and, as explained in response to Reviewer 1, we have now made this more explicitly stated in the introduction and discussion.

Study design is appropriate to this model development, the population and data set were clearly described with the "sample size" of blocks being clearly described and justified (no clear sample size calculation, seems to be based on availability rather than statistically driven number of blocks needed to make the best model; while perhaps not ideal, this is a real-world application and I think appropriate methodology was used in this paper).

A sample size calculation was not appropriate in this context since we were not sampling from a population (interpreting “population” here as VL-affected sub-districts) in order to test a particular hypothesis. This is the most comprehensive dataset of incidence in the endemic region and therefore we have essentially measured the whole “population” of sub-districts. There were some additional data from peripheral regions (e.g in the neighbouring states of West Bengal and Uttar Pradesh) but these were known to be incomplete. We therefore defined our study area as the region of Bihar and Jharkhand widely accepted as being most heavily-affected, and considered that as our “population” of interest.

No concern about the statistical tests to compare the models as described. No concerns regarding the ethical requirements.

--------------------

*Results*

Reviewer #1: The lack of reference to previous studies makes difficult the comparison of the forecasts, since measurements like the reduction in the mean square error (or predictive r squared) are not presented.

As explained above (from line 133), multiple sources advocate the use of scoring rules (Gneiting (2014), Lu (2020), Funk (2020)) as an informative measure of predictive power, with benefits over the more traditional metric of absolute or squared error. For comparison, we have now presented the MAE in addition to the RPS for the final model (line 338). We do not, however, advise direct comparison of such measures across studies based in different contexts with different data sources.

lines 252-256: Seems it will be best to try zero inflated count models for those districts.

We thank the referee for this suggestion. Zero-inflated models are mentioned in the discussion (line 483). Also, a spatial model such as this requires the same likelihood for all spatial units, therefore it would not be possible to fit a ZI model for a subset of districts. However, we are actively pursuing alternative approaches in which ZI are being considered.

Line 483: *The variation in case counts may be better explained by a zero-inflated process, and the extent of zero-inflation will likely become more prominent as elimination is approached.*

Table s1 only shows what seems like a rather small subsample of the models explored (looking at the model numbers).

Indeed – the table includes only the models which were selected at each stage, as was stated on line 307 of the original manuscript. For clarity, we have now included the full table of models in the supplementary materials, and amended the description of this table:

Line 295: Fit and prediction metrics for all models are given in Table S1 and PIT histograms for the models selected at each stage are compared in Fig. S3.

Reviewer #2: The analysis matches what was planned in methods well. The results are described well in the text with graphics to help illustrate (though some of these are less helpful to the average reader and could be made supplemental (such as Figures 5 and 6). Overall very readable for a very densely mathematical paper. The authors start to overlap into discussion a little in the results section (e.g., talking about limitations of the models produced).

We have now moved the suggested figures to the supplementary material and rearranged the text to remove the point about model limitations from the results section to the discussion (line 474).

Line 747: There is clearly a limitation of fitting such a model over a large number of highly heterogeneous units with minimal unit-specific information.

--------------------

*Conclusions*

Reviewer #1: lines 282-285, this conclusion might be an artifact of the non-systematic model selection.

We took a systematic model selection approach to reach this conclusion during the preliminary model evaluation (line 264 of the original manuscript, now moved to Appendix B “Distributed Temporal Lags”). However, we are still unsure about the explanation for this finding and it is an on-going subject for our research. We currently believe that this is something to do with shifting variance in predictions between the average outcomes and the underlying distribution. It could also be due to interactions between the incubation period and reporting delays. We stopped at four month lags as the improvement plateaus at that point. We could show results for five month lags but they are not materially different.

Reviewer #2: Conclusions are supported by the data, and between here and the results section the limitations of the model are well described (such as model driven by low incidence blocks and that areas with higher cases or sporadically high case burden were less well predicted). They also very clearly stated that this model would be limited if incidence continues to fall off and that the approach needs to be broadened to a more regional approach that is not artificially bounded by country borders. They very practically designed the model to meet the needs of the elimination program with consideration of data delay built in trying to balance practicality with predictability. This is very relevant and applicable to the real world situation program in Southern Asia.

Thank you.

--------------------

*Editorial and Data Presentation Modifications*

Reviewer #1: Be more careful when compiling your latex files in line 209 (and others) the "<" was written as "!"

We thank the reviewer for pointing this error out - it has now been corrected.

Reviewer #2: Overall very well written and understandable language used.

--------------------

*Summary and General Comments*

Reviewer #1: This study is on an interesting topic, though it shows very preliminary results and fails to put in context the results in light of what has been done previously for leishmaniasis forecasting. Surprisingly no effort was made to test whether meteorological information improved the forecasts. One major issue is that the analysis was very constrained based on what was implemented in the R package used to fit the models.

We have expanded our introduction with additional references to previous studies of leishmaniasis forecasting in response to earlier comments. We acknowledge in the discussion (lines 465-468) that geographical/meteorological data may explain some of the heterogeneity that the current model cannot account for, and we are attempting to obtain such data for future work. The intention of this study was to ascertain the potential utility of predictions based solely on routinely-collected surveillance data, within a ready-made, rapid and relatively easy-to-use framework which could be routinely implemented in-country. We have made this more explicit in the hypothesis on line 169.

The study uses a relatively novel way to study time series, yet the methods are described in a highly mystified way, for the general audience of PLoS NTDs.

We have moved a portion of section 2.3.1 (detail on empirical coverage) and section 3.1 (preliminary analysis of dispersion and temporal lags) into the supplementary material so that the main body of the paper focusses on practical application rather than technical detail. Whilst we realise that paper is relatively technical (although see papers by Chaves (2007), Paixao-Seva (2017) and Lewnard (2014) suggested above by the reviewer with similar technical detail). VL is an important NTD; we propose a novel approach to aid its elimination in India, and it is necessary to fully define the approach.

Reviewer #2: Overall very good paper with an aim to be practical. As above there are a few more technical figures that can be added to the supplementary material and some of the results would be more ideally incorporated in the discussion as many readers practically will read the abstract then the discussion and then if interested go back to the results and maybe the methodology so for those readers they will miss some of the very valuable discussion on the limitations and why those were chosen and the practical approach the authors took to develop this model. Just a suggestion though as overall a very good paper.

We hope that these amendments to the manuscript are satisfactory to address the questions and comments raised.

Kind regards,

Emily Nightingale
