# Supplementary figures and images for: Correction: A spatio-temporal approach to short-term prediction of visceral leishmaniasis diagnoses in India
Source: PLoS Negl Trop Dis. 2022 Apr 4;16(4):e0010346. doi: 10.1371/journal.pntd.0010346 (PMC8979458; doi:10.1371/journal.pntd.0010346)

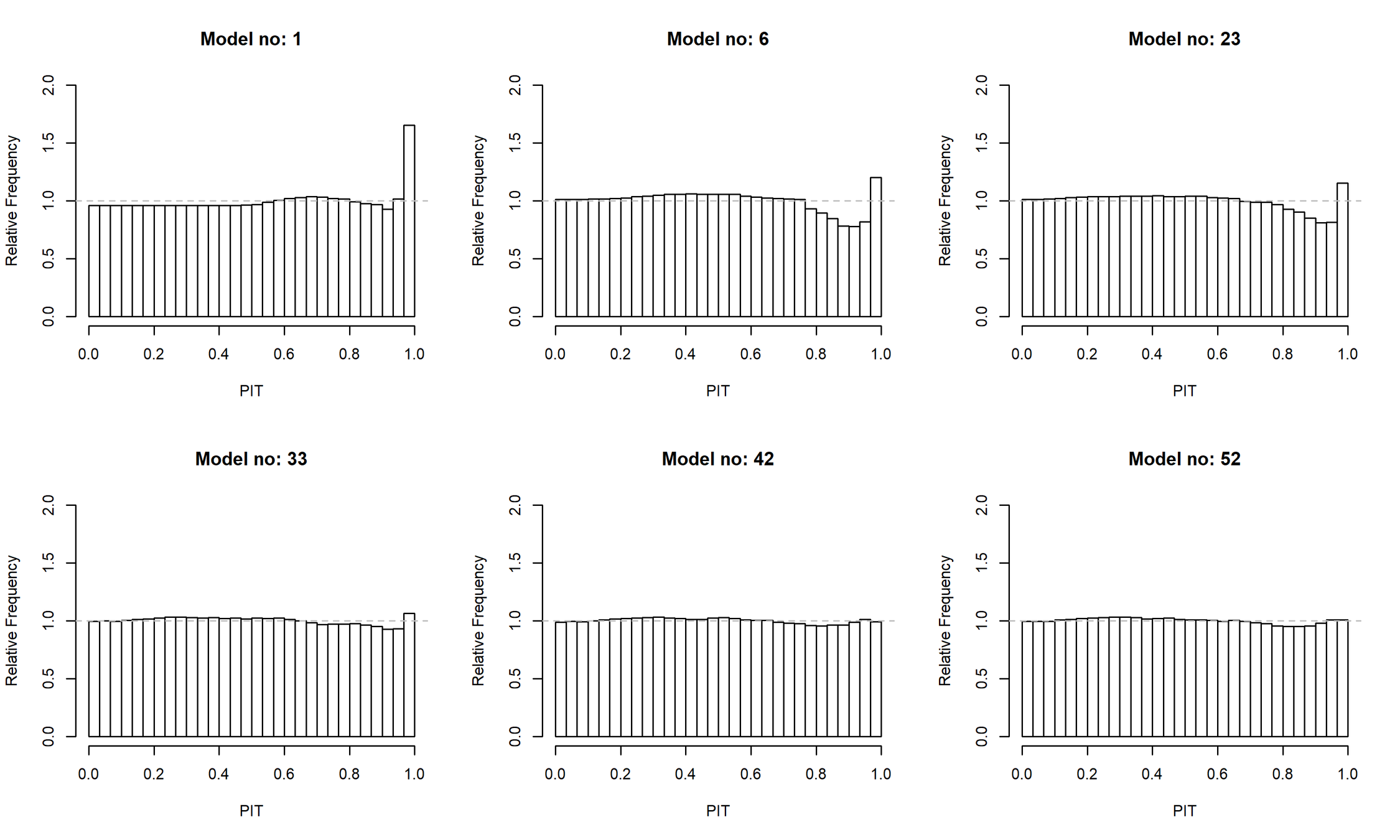

Supplement: S3 Fig — Model 42 is the final model. Model 52 offered minor improvement in RPS with additional complexity. (TIF) [file pntd.0010346.s001.tif]
